# DNA End Resection: Facts and Mechanisms

**DOI:** 10.1016/j.gpb.2016.05.002

**Published:** 2016-05-27

**Authors:** Ting Liu, Jun Huang

**Affiliations:** 1 Department of Cell Biology and Program in Molecular Cell Biology, Zhejiang University School of Medicine, Hangzhou 310058, China; 2 Life Sciences Institute and Innovation Center for Cell Signaling Network, Zhejiang University, Hangzhou 310058, China

**Keywords:** DNA end resection, Homologous recombination, DNA double-strand breaks, Chromatin remodeling factors, Genome stability

## Abstract

**DNA double-strand breaks** (DSBs), which arise following exposure to a number of endogenous and exogenous agents, can be repaired by either the **homologous recombination** (HR) or non-homologous end-joining (NHEJ) pathways in eukaryotic cells. A vital step in HR repair is **DNA end resection**, which generates a long 3′ single-stranded DNA (ssDNA) tail that can invade the homologous DNA strand. The generation of 3′ ssDNA is not only essential for HR repair, but also promotes activation of the ataxia telangiectasia and Rad3-related protein (ATR). Multiple factors, including the MRN/X complex, C-terminal-binding protein interacting protein (CtIP)/Sae2, exonuclease 1 (EXO1), Bloom syndrome protein (BLM)/Sgs1, DNA2 nuclease/helicase, and several chromatin remodelers, cooperate to complete the process of end resection. Here we review the basic machinery involved in **DNA end resection** in eukaryotic cells.

## Introduction

Double-strand breaks (DSBs) are one of the most dangerous types of DNA damage because they disrupt the continuity of chromosomes [1], [2]. Failure to eliminate DSBs leads to genome instability and tumorigenesis [1], [3]. DSBs are predominantly repaired by either the non-homologous end-joining (NHEJ) pathway or the homologous recombination (HR) pathway [4], [5]. NHEJ directly ligates the broken DNA ends, whereas HR uses a homologous sequence from sister chromatid as a repair template [4], [6].

Using an identical or similar sequence as a template, HR is commonly considered to be an error-free mechanism for the repair of DSBs [7], [8]. When DSBs occur, a process termed DNA end resection is activated, which catalyzes the nucleolytic degradation of the broken ends in the 5′ to the 3′ direction [9], [10]. The resulting 3′ single-stranded DNA (ssDNA) then provides a platform for the recruitment of proteins that participate in HR repair [9], [10], [11]. Interestingly, DNA end resection inhibits NHEJ and triggers homology-directed DSB repair [11]. Multiple proteins or protein complexes have been shown to be involved in this process. These include the MRN complex (MRX complex in budding yeast), C-terminal-binding protein interacting protein (CtIP; Sae2 in budding yeast), exonuclease 1 (EXO1), Bloom syndrome protein (BLM; Sgs1 in budding yeast), DNA2 nuclease/helicase, and several chromatin remodeling factors [12]. Here, we discuss the pivotal proteins and their mechanisms during DNA end resection.

## DNA end resection and the repair pathway choice

Although DSBs can occur at any phase of the cell cycle, DNA end resection only happens in the S and G2 phases [9], [13]. During other cell cycle phases, DNA end resection is inhibited by Ku70/80 heterodimers and other proteins; therefore, only the NHEJ pathway can be initiated [11]. NHEJ promotes direct ligation of the DNA ends; subsequent processing of the broken DNA ends is unnecessary [11]. This phenomenon is also consistent with the finding that sister chromatids only exist in the S and G2 phases. However, the repair pathway choice also depends on substrate complexity and other factors besides the cell cycle [13], [14].

## The MRN/X complex

The MRN complex, which comprises MRE11, RAD50, and nibrin (NBS1), plays key roles in DNA end resection and HR repair in mammalian cells [15], [16]. The counterpart of the MRN complex in budding yeast is the MRX complex, which consisting of Mre11, Rad50, and Xrs2 [17]. The MRN/X complex not only functions in DNA end resection, but also plays critical roles in the DNA damage checkpoint response [18].

The MRN complex binds DNA through its globular domain, in which MRE11 and NBS1 associate with the Walker A and Walker B motifs of RAD50 [19]. Previous studies suggest that the DNA binding activity requires primarily MRE11 and RAD50 [19], [20], [21]. The extended coiled-coil tail of RAD50 forms another structural domain in the whole MRN complex, which is important for the DNA-binding and -tethering activities of the complex [22], [23], [24], [25], [26].

MRE11 is the core component of the MRN complex and exhibits a variety of enzymatic activities, including 3′ to 5′ exonuclease activity on dsDNA, endonuclease activity on ssDNA, and DNA-annealing and -unwinding activities [20], [21], [27], [28]. *In vitro* experiments revealed that the five phosphoesterase motifs within the N-terminal region of MRE11 are essential for its biochemical activities [12], [29]. Paradoxically, generation of the 3′ overhang requires the activity of 5′ to 3′ exonuclease, which is opposite to the observed exonuclease activity of MRE11 [30], [31]. A two-step mechanism of MRE11 has thus been proposed, that is, MRE11 makes the initial ssDNA nick via its ssDNA endonuclease activity at first and then digests toward the DSB end through its 3′ to 5′ exonuclease activity to produce 3′ ssDNA tails [31], [32], [33], [34].

Human NBS1 contains two BRCA1 C terminus (BRCT) domains and a forkhead-associated (FHA) domain [35]. Mutations within the *NBS1* gene are responsible for the Nijmegen breakage syndrome, a rare autosomal recessive disease that increases the predisposition to develop malignancies [36], [37], [38]. Cells derived from NBS patients exhibit defects in DSB repair and cell cycle checkpoint [36]. Although lacking enzymatic activities, NBS1 is considered to be an important regulator in the MRN complex, since NBS1 influences both DNA binding and nuclease activity of MRE11 [39], [40], [41].

## CtIP/Sae2

CtIP was first identified as a cofactor for the transcriptional repressor C-terminal-binding protein (CtBP) [12], [42]. Further studies reveal that CtIP functions in many other cellular processes, including cell cycle regulation and tumorigenesis [43]. Interestingly, CtIP is now better known as an interacting partner of the MRN complex, for its involvement in DNA end resection and DSB repair [44], [45], [46], [47].

CtIP shows sequence homology to the budding yeast Sae2 at the C terminus [48]. CtIP plays at least two roles in the process of DNA end resection, distinguished by the involvement of its catalytic activity or not [49]. Briefly, the resection of DSBs with clean broken ends produced by restriction enzymes is dependent on the presence of CtIP protein, but independent of its nuclease activity [49], [50]. By contrast, the repair of more complex DNA lesions created by topoisomerase poisons or ionizing radiation (IR) requires not only the presence of CtIP protein but also its endonuclease activity [51].

## EXO1

EXO1 belongs to the xeroderma pigmentosum complementation group G (XPG) family of nucleases, which contain conserved nuclease motifs in the N-terminal region [52], [53]. EXO1 exhibits 5′ to 3′ dsDNA exonuclease and 5′ flap endonuclease activities *in vitro* [12], [53]. Interestingly, EXO1 prefers dsDNA substrates with a recessed 5′ end, which is produced by the MRN/X complex and CtIP/Sae2 [54], [55], [56]. Taken together with the finding that MRE11 lacks the 5′ to 3′ exonuclease activity required to produce long 3′ ssDNA tails necessary for replication protein A (RPA) binding, a two-step model has been suggested for DSB processing [12]. In this model, the MRN/X complex and CtIP/Sae2 remove the first 50–100 nucleotides from the 5′ end of the broken DNA, followed by the generation of long 3′ ssDNA tails catalyzed by EXO1 [12]. This model is also supported by the finding that CtIP is required for the accumulation of EXO1 at DSB sites *in vivo* [57].

## DNA2–BLM/Sgs1

BLM is a member of the RecQ family of helicases that unwinds DNA in mammals and Sgs1 is its ortholog in *Saccharomyces cerevisiae* [58]. DNA2, which is related to the bacterial RecB proteins, exhibits both helicase and nuclease activities *in vitro* [33]. However, the helicase activity of DNA2 is not necessary for DNA end resection, while the nuclease activity of DNA2 is essential to this process [59], [60], [61]. Previous reports suggest that EXO1 and DNA2–BLM/Sgs1 function in parallel at the second step of end resection [12]. Interestingly, studies in yeast indicate that Sgs1-Dna2-catalyzed end resection is dependent on RPA [61]. In the absence of RPA, DNA2 cannot be recruited to DSBs [61]. Although it can degrade either 3′- or 5′-terminated ssDNA, DNA2 exhibits 5′ endonuclease activity only in the presence of RPA, which may explain the strand bias in the end resection [61].

## Chromatin remodeling factors

**Figure 1:**
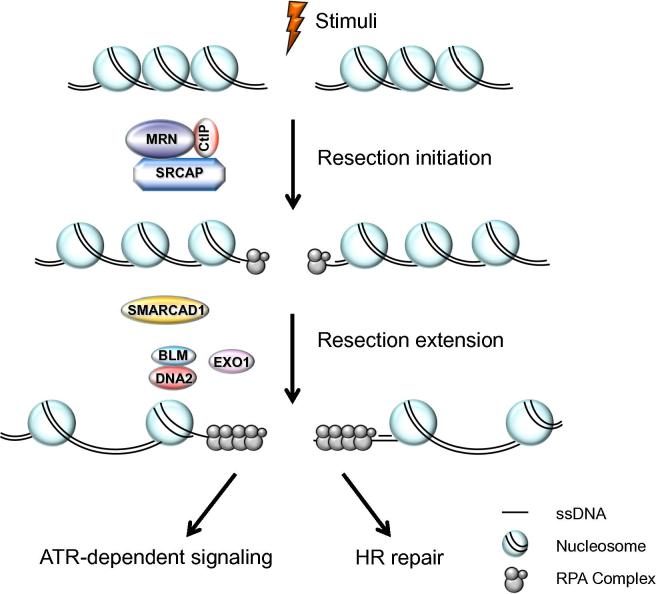
DNA-end resection occurs via a two-step process—resection initiation and resection extension Resection initiation is stimulated by SRCAP, CtIP, and the MRN complex. SMARCAD1 cooperates with EXO1 and BLM/DNA2 to promote resection extension. The figure is adapted from [70]. ATR, ataxia telangiectasia and Rad3-related protein; BLM, Bloom syndrome protein; CtIP, C-terminal-binding protein interacting protein; EXO1, exonuclease 1; HR, homologous recombination; MRN, MRE11, RAD50, and nibrin; RPA, replication protein A; SMARCAD1, switch/sucrose non-fermentable (SWI/SNF)-related matrix-associated actin-dependent regulator of chromatin subfamily A containing DEAD/H box 1; SRCAP, Snf2-related CREB-binding protein activator protein; ssDNA, single-stranded DNA.

Eukaryotic DNA is normally wrapped around a histone octamer to form nucleosomes [62]. This condensation allows the long genetic molecules to fit into the relatively-small nucleus, but at the same time, forms a barrier for resection enzymes to access [63]. Certain histone modifiers, histone chaperones, and chromatin remodelers modify chromatin structure and hence regulate the dynamics of the chromatin [64], [65]. For instance, several chromatin remodeling factors, such as remodels the structure of chromatin (RSC), INO80, switch/sucrose non-fermentable (SWI/SNF), SWI/SNF-related matrix-associated actin-dependent regulator of chromatin subfamily A containing DEAD/H box 1 (SMARCAD1; Fun30 in yeast), and Snf2-related CREB-binding protein (CREBBP) activator protein (SRCAP; SWR1 in yeast), are involved in the process of overcoming barriers to allow repair proteins to access (Figure 1) [66], [67], [68], [69], [70], [71].

Rad9, the yeast 53BP1 ortholog, is a checkpoint mediator protein and is recruited to DSB sites by γ-H2A and K79-methylated histone H3 [72]. Rad9 is known to inhibit DNA end resection [72]. On the other hand, the yeast Fun30, which possesses intrinsic ATP-dependent chromatin remodeling activity, works together with DNA2 and EXO1 to promote extensive DSB end resection [71], [73], [74], [75]. Mechanically, Fun30 overcomes the barrier formed by Rad9-bound chromatin, thus promoting extensive resection process [71], [73], [74], [75].

SRCAP is a member of the INO80 ATPase family and belongs to the human SRCAP chromatin remodeling complex [76]. SRCAP was first discovered as the binding partner of CREBBP (also known as CBP), and mutations in SRCAP cause a rare genetic disorder known as Floating–Harbor syndrome [76], [77]. Our recent findings support a new function for SRCAP in promoting DSB resection and HR repair (Figure 1). SRCAP-depleted cells exhibit RPA2 hyperphosphorylation and defects in RPA2 focus formation, indicating that SRCAP is involved in DSB end processing [78]. Accordingly, SRCAP depletion only affects IR- and camptothecin (CPT)-induced RPA2 focus formation but not hydroxyurea (HU)-induced RPA2 focus formation (HU stalls replication forks by deprivation of dNTPs) [78]. Moreover, zinc finger HIT-type 1 (ZNHIT1)/p18, another component of the SRCAP complex, also promotes DNA end resection [78]. Mechanistically, SRCAP promotes chromatin relaxation to allow CtIP accumulation at DSB sites, thereby facilitating DSB end processing (Figure 1) [78].

## Summary

Correct repair of DSBs is critical for the maintenance of genome stability. HR and NHEJ are the two dominant repair pathways involved in DSB repair [4], [6]. While NHEJ facilitates the direct ligation of the DSB ends in an error-prone manner, HR allows for precise repair of DSBs due to the employment of homologous chromatids [8]. DNA end resection is a pivotal step in HR repair to produce 3’ overhangs that not only inhibit NHEJ but also provide a platform to recruit proteins involved in HR repair [11]. DNA end resection is completed through a two-step process in which the MRN/X complex and CtIP/Sae2 protein are involved in the initial step, and EXO1 and DNA2-BLM/Sgs1 are involved in the second step [79]. However, precisely how these resection factors are regulated in a coordinated manner is still unclear. Further studies are therefore required to resolve this issue.

## Competing interests

The authors declare no competing financial interests.
